# Granulomatosis With Polyangiitis Presenting With H1N1 Influenza

**DOI:** 10.7759/cureus.98284

**Published:** 2025-12-02

**Authors:** Ahmed Raza, Ahmed Algahiny

**Affiliations:** 1 Pulmonology, University Hospitals of Leicester NHS Trust, Leicester, GBR; 2 Internal Medicine, University Hospitals of Leicester NHS Trust, Leicester, GBR

**Keywords:** antineutrophil cytoplasmic antibody (anca), autoimmune vasculitis, granulomatosis with polyangiitis (gpa), h1n1 influenza, organising pneumonia

## Abstract

Granulomatosis with polyangiitis (GPA) is a rare antineutrophil cytoplasmic antibody (ANCA) associated vasculitis characterized by necrotizing inflammation of small- to medium-sized vessels, most commonly affecting the respiratory tract and kidneys. We report the case of a 74-year-old man who presented with H1N1 influenza infection followed by renal failure. Serologic testing revealed elevated PR3-ANCA, and renal biopsy confirmed a pauci-immune vasculitis consistent with a diagnosis of GPA requiring immunosuppressive therapy with corticosteroids and rituximab. This case highlights the necessity of a thorough evaluation for autoimmune aetiologies in patients presenting with unexplained pulmonary symptoms.

## Introduction

Granulomatosis with polyangiitis (GPA), previously known as Wegener's granulomatosis, is a necrotizing vasculitis that affects small to medium-sized blood vessels, leading to inflammation and tissue damage. It belongs to a group of rare autoimmune conditions collectively referred to as antineutrophil cytoplasmic antibody (ANCA)-associated vasculitis, which are characterized by inflammation of small to medium-sized vessels and present with a range of clinical manifestations [1]. The three main types of ANCA-associated vasculitis are GPA, microscopic polyangiitis, and eosinophilic granulomatosis with polyangiitis (formerly Churg-Strauss syndrome) [1]. Because GPA can involve several organs at once, its early symptoms are often non-specific and may resemble common illnesses. It commonly involves the respiratory tract, kidneys, nasal septum, and sinuses [2]. Renal involvement is seen in approximately 50% of patients at the time of diagnosis, typically presenting with reduced kidney function, proteinuria, and haematuria [3]. Respiratory features such as sinus pain, nasal congestion, cough, and general flu-like symptoms are common initial complaints. Untreated GPA can progress rapidly and cause permanent organ damage, so timely recognition is essential. We present a case of a patient who initially presented with symptoms suggestive of an upper respiratory tract infection and was diagnosed with H1N1. The patient subsequently deteriorated, required admission to the intensive care unit (ICU), and was eventually diagnosed with GPA.

## Case presentation

A 74-year-old Caucasian male presented with a one-week history of coryzal symptoms, haemoptysis, and chest pain. His past medical history includes hypertension, and he had a good baseline performance status (Eastern Cooperative Oncology Group 1), remaining fully active and cycling twice weekly. He was admitted to the ICU with low oxygen saturations and low Glasgow Coma Scale (GCS) requiring intubation, and was successfully decannulated after three days.

Initial investigations revealed markedly elevated inflammatory markers, including C-reactive protein and white cell count, suggesting a significant systemic inflammatory response. A bronchoalveolar lavage (BAL) was positive for H1N1 influenza. Microbiological testing, including fungal cultures, tuberculosis screening, and microscopy, was negative. Despite multiple sets of blood cultures, no bacterial growth was identified. Procalcitonin was also elevated, supporting a possible mixed infectious and inflammatory etiology.

**Figure 1:**
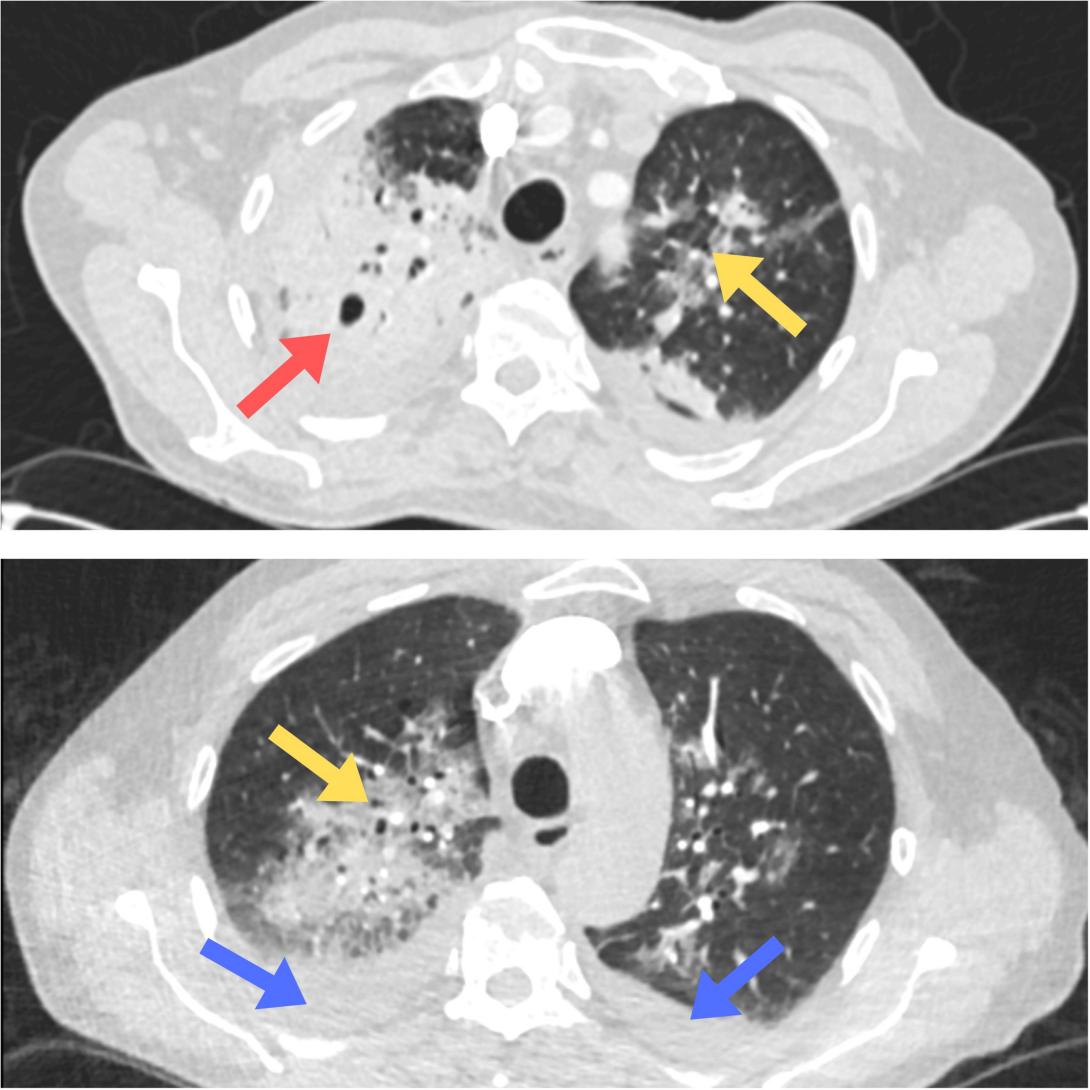
CT Thorax showing multifocal bilateral pneumonic infiltrates, bilateral pleural effusions, and organizing pneumonia Red arrow showing cavitatory lesion in right lung, yellow arrow showing consolidation. Blue arrows showing bilateral pleural effusions.

Chest X-ray showed patchy bilateral consolidations. CT Thorax showed multifocal bilateral pneumonic infiltrates, bilateral pleural effusions, and radiological features consistent with organizing pneumonia, as shown in Figure 1. Cardiac evaluation revealed new-onset atrial fibrillation on ECG, likely sepsis-driven. Transthoracic echocardiogram revealed severe cardiac dysfunction, including a significantly dilated left ventricular cavity with a severely impaired ejection fraction of 35% and severe functional mitral regurgitation. The study also noted a markedly dilated left atrium, a dilated right atrium, and signs suggestive of pulmonary hypertension. N-terminal prohormone of brain natriuretic peptide (NT-proBNP) levels were significantly raised, further supporting the diagnosis of acute decompensated heart failure. A CT scan of the abdomen and pelvis showed no evidence of intra-abdominal pathology.

The patient was treated with antivirals (Tamiflu) followed by multiple courses of antibiotics, including co-amoxiclav, tazocin, vancomycin, clarithromycin, co-trimoxazole, and teicoplanin (post-biopsy). He was anticoagulated with apixaban and rate-controlled with bisoprolol for atrial fibrillation. For heart failure, he was managed with furosemide, spironolactone, and Entresto. 

Kidney functions were closely monitored, and the patient experienced sudden renal deterioration with a significant fall in eGFR, rising creatinine, new haematuria, and substantial proteinuria, developing acute kidney injury stage 3. This accelerated trajectory was clinically notable and raised concern for a systemic process beyond infection. An autoimmune screen revealed a high proteinase 3 (PR3)-ANCA and rheumatoid factor, while other autoantibodies, including C-ANCA, MPO-ANCA, antinuclear antibody (ANA), extractable nuclear antigen (ENA), anti-GBM, anti-cyclic citrullinated peptide (CCP), cardiolipin, and myositis profile were negative. A summary of the investigation workup is shown in Table 1. Renal biopsy confirmed necrotising crescentic glomerulonephritis with pauci-immune features, consistent with ANCA-associated vasculitis.

**Table 1:** Summary of initial investigations eGFR - estimated glomerular filtration rate; NT-proBNP - N-terminal prohormone of brain natriuretic peptide; PR3 - proteinase 3; ANCA - antineutrophil cytoplasmic antibody

Investigation	Value	Reference range
Procalcitonin	1.36 ng/mL	< 0.05 ng/mL
NT-proBNP	13,504 ng/L	< 400 ng/L
eGFR	19 ml/min	>90 ml/min
Creatinine	293 umol/L	59 - 104 μmol/L
Urine albumin to creatinine ratio	313 mg/mmol	< 15 mg/mmol
Random urinary protein	1.69 g/L	< 0.15 g/L
PR3-ANCA	179.2 IU/mL	< 2 IU/mL
Rheumatoid factor	23 IU/mL	< 20 IU/mL

**Figure 2:**
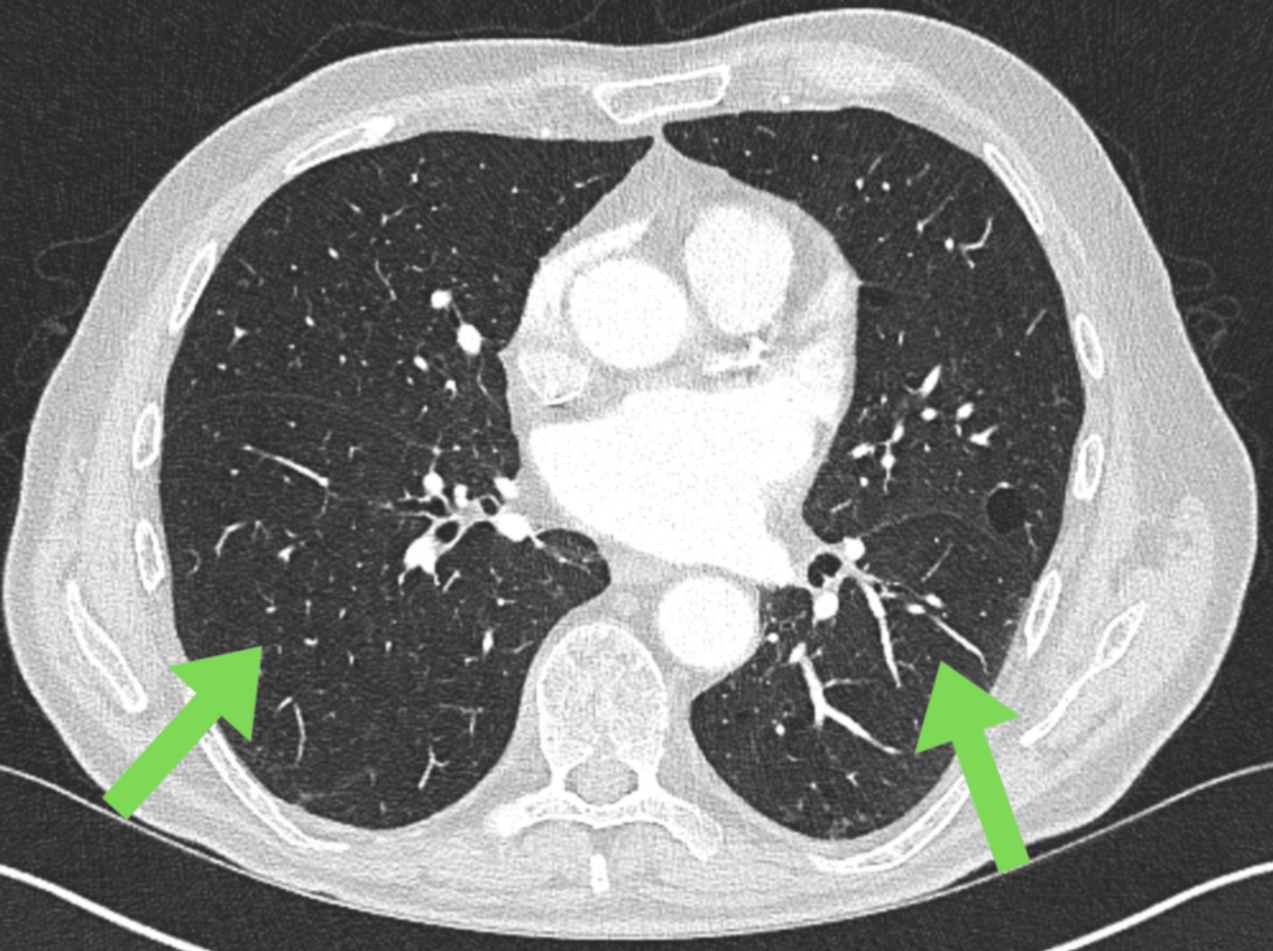
Clear CT thorax with resolution of previous findings Green arrows showing interval resolution of cavitatory and consolidatory lesions with resolution of effusion.

Immunosuppressive therapy included intravenous methylprednisolone for three days followed by a tapering course of prednisolone. He received two doses of rituximab for ANCA-associated vasculitis. Avacopan, a complement 5a receptor antagonist, was initiated but later paused due to elevated liver enzymes (ALT). He is currently being followed up jointly in the interstitial lung disease (ILD) and renal multidisciplinary team clinic. The three-month follow-up and repeat CT thorax showed resolution of the pneumonia and pleural effusion, as shown in Figure 2.

## Discussion

GPA is a rare systemic necrotizing vasculitis that primarily affects small to medium-sized blood vessels [1]. It characteristically involves the upper and lower respiratory tract and the kidneys, often presenting with a triad of sinusitis, pulmonary infiltrates, and glomerulonephritis. The pathogenesis involves granulomatous inflammation and is strongly associated with ANCA, particularly PR3-ANCA. Renal involvement typically manifests as rapidly progressive glomerulonephritis with hematuria and proteinuria, while pulmonary features include infiltrates, hemoptysis, and, in some cases, alveolar haemorrhage [4]; however, up to one-third of patients do not have hemoptysis [5]. Without prompt treatment, GPA can progress rapidly and be life-threatening, but with early diagnosis and immunosuppression, the prognosis significantly improves [6].

Viral infections such as hepatitis C virus, cytomegalovirus, Epstein-Barr virus, and parvovirus are recognized triggers for GPA [7,8,9]. Although no studies have demonstrated a direct association between H1N1 and GPA, one case report identified H1N1 at the time of the initial GPA diagnosis, similar to our patient [10]. This finding suggests that H1N1 may represent a potential trigger for GPA.

In our patient, initial presentation with coryzal symptoms, hemoptysis, and hypoxic respiratory failure was attributed to H1N1 influenza infection, confirmed on bronchoalveolar lavage. The clinical picture was initially suggestive of severe viral pneumonia complicated by acute respiratory distress syndrome (ARDS), requiring intensive care support and intubation. However, over the following days, the emergence of additional systemic findings, specifically acute kidney injury with haematuria and proteinuria, positive PR3-ANCA, and severe cardiac dysfunction, prompted reconsideration of the diagnosis. The suspicion of GPA became more prominent when renal function rapidly deteriorated, along with positive serological testing (PR3-ANCA), prompting a renal biopsy. Histopathology confirmed necrotizing crescentic glomerulonephritis with pauci-immune features, solidifying the diagnosis of ANCA-associated vasculitis, most consistent with GPA.

The diagnostic process involved a wide array of investigations. Infectious causes were comprehensively evaluated, including fungal, mycobacterial, and bacterial cultures, which all returned negative. Despite elevated procalcitonin, there was no bacteremia identified, suggesting a significant inflammatory component likely amplified by the H1N1 infection. Procalcitonin is typically not elevated during autoimmune flare-ups but expected to be high in bacterial infections, with high sensitivity and specificity of 81.3% and 78.7% respectively, as reported by Joo et al. [11], making it a useful marker for differentiation [12,13]. Herrmann et al. also reported a specificity of 92% and a sensitivity of 60% [13]. Nonetheless, elevated levels have occasionally been reported in autoimmune conditions even without superimposed bacterial infection [12]. For instance, Moosig et al. reported average procalcitonin levels of 0.8-3.3 ng/ml in highly active GPA [14].

Imaging studies revealed bilateral pulmonary infiltrates and pleural effusions with radiological features consistent with organizing pneumonia. Cardiac investigations revealed new-onset atrial fibrillation, severe left ventricular dysfunction, functional mitral regurgitation, and pulmonary hypertension, further complicating the clinical course. Serological evaluation was pivotal, with a markedly raised PR3-ANCA and elevated rheumatoid factor, while all other autoimmune markers, including anti-GBM antibodies, ANA, and MPO-ANCA, were negative. The renal biopsy was ultimately decisive, confirming the autoimmune etiology.

A broad differential was initially considered. Infectious pneumonia, viral acute respiratory distress syndrome (ARDS), and secondary bacterial superinfection were high on the list, particularly in the context of influenza. Given the pulmonary-renal involvement, microscopic polyangiitis (MPA) and anti-GBM disease were also considered. However, the serological profile (PR3-ANCA positivity) and the absence of anti-GBM antibodies made GPA more likely. Additionally, organizing pneumonia, a finding on CT, may have been secondary to the underlying vasculitic process or post-viral inflammation.

The patient received comprehensive treatment tailored to his complex presentation. Initially, he was stabilized with broad-spectrum antibiotics, antivirals, and supportive care for heart failure and atrial fibrillation. Once autoimmune vasculitis was suspected, high-dose intravenous methylprednisolone was initiated, followed by a weaning course of oral prednisolone. Rituximab was administered as induction therapy in accordance with current guidelines for organ-threatening ANCA-associated vasculitis. Avacopan for ANCA vasculitis was also initiated but later paused due to elevated liver enzymes. Management of heart failure included loop diuretics, spironolactone, and sacubitril/valsartan (Entresto), while anticoagulation and rate control were achieved with apixaban and bisoprolol, respectively. His care was coordinated through a multidisciplinary team involving nephrology, pulmonology, and cardiology. On the latest review of the eGFR, creatinine and urine albumin/creatinine ratio have normalised. 

## Conclusions

This case highlights the critical importance of considering autoimmune conditions like GPA in patients with multi-organ dysfunction, particularly when features such as haematuria, pulmonary infiltrates, and systemic inflammation are present. Infections may mask or trigger autoimmune processes, complicating the diagnosis. Prompt recognition of autoimmune vasculitis is essential, as delayed treatment can lead to irreversible organ damage and increased mortality. The presence of PR3-ANCA, a hallmark of GPA, combined with renal biopsy findings, was essential in confirming the diagnosis and initiating life-saving immunosuppressive therapy.

## References

[1] Jennette JC, Falk RJ, Bacon PA (2013). 2012 revised International Chapel Hill Consensus Conference Nomenclature of Vasculitides. Arthritis Rheum.

[2] Polychronopoulos VS, Prakash UB, Golbin JM, Edell ES, Specks U (2007). Airway involvement in Wegener's granulomatosis. Rheum Dis Clin North Am.

[3] Kronbichler A, Shin JI, Lee KH (2020). Clinical associations of renal involvement in ANCA-associated vasculitis. Autoimmun Rev.

[4] Mahajan V, Whig J, Kashyap A, Gupta S (2011). Diffuse alveolar hemorrhage in Wegener's granulomatosis. Lung India.

[5] Thickett DR, Richter AG, Nathani N, Perkins GD, Harper L (2006). Pulmonary manifestations of anti-neutrophil cytoplasmic antibody (ANCA)-positive vasculitis. Rheumatology (Oxford).

[6] Gavica JC, Raymond L (2023). Significance of early treatment in granulomatosis with polyangiitis vasculitis. Clin Case Rep.

[7] Rout P, Garlapati P, Qurie A (2025). Granulomatosis With Polyangiitis. https://www.ncbi.nlm.nih.gov/books/NBK557827/.

[8] Nikkari S, Mertsola J, Korvenranta H, Vainionpää R, Toivanen P (1994). Wegener's granulomatosis and parvovirus B19 infection. Arthritis Rheum.

[9] Gayen S, Zhang D, Sternlicht E, Bulanowski D, Tabba M (2022). Granulomatosis with polyangiitis: the trigger cannot be long hidden. Eur J Rheumatol.

[10] Monteiro M, Domingos R, Rocha S, Miranda I (2023). Granulomatosis with polyangiitis: The complexity of clinical manifestations, therapeutic challenges, and complications of a severe multisystemic case. Cureus.

[11] Joo K, Park W, Lim MJ, Kwon SR, Yoon J (2011). Serum procalcitonin for differentiating bacterial infection from disease flares in patients with autoimmune diseases. J Korean Med Sci.

[12] Buhaescu I, Yood RA, Izzedine H (2010). Serum procalcitonin in systemic autoimmune diseases--where are we now?. Semin Arthritis Rheum.

[13] Herrmann K, Schinke S, Csernok E, Moosig F, Holle JU (2015). Diagnostic value of procalcitonin in ANCA-associated vasculitis (AAV) to differentiate between disease activity, infection and drug hypersensitivity. Open Rheumatol J.

[14] Moosig F, Csernok E, Reinhold-Keller E, Schmitt W, Gross WL (1998). Elevated procalcitonin levels in active Wegener's granulomatosis. J Rheumatol.

